# Supplementary figures and images for: Sequoia affects Drosophila central nervous system development by regulating axonal extension and guidance
Source: PLoS One. 2026 Mar 25;21(3):e0333573. doi: 10.1371/journal.pone.0333573 (PMC13016347; doi:10.1371/journal.pone.0333573)

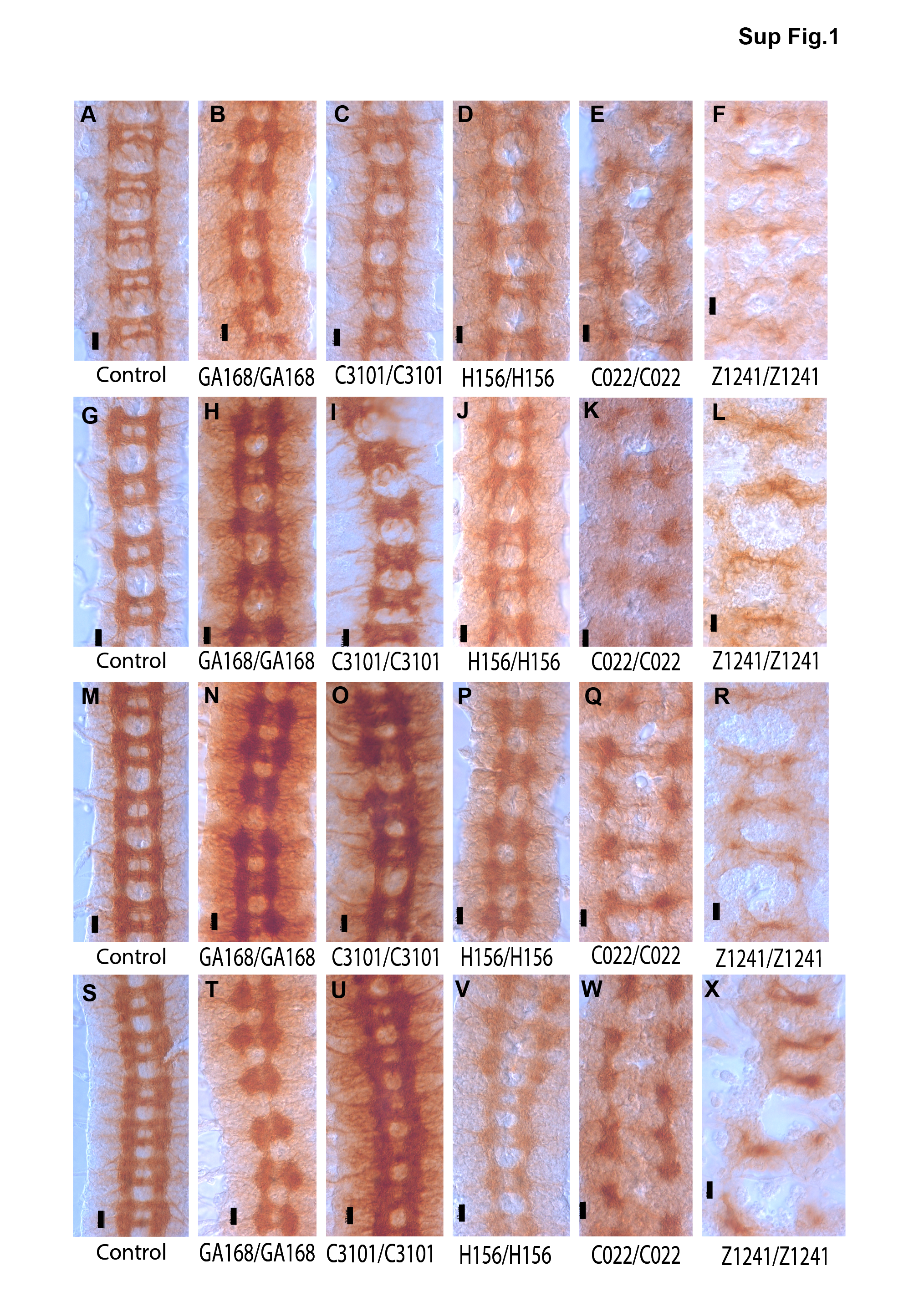

Supplement: S1 Fig — All these embryos were stained with BP102, which targets anti-CNS axons, and examined during development. (A) At developmental stages 13–14, the two commissural tracts separate, and the longitudinal tracts begin to develop at the commissural points. (B) In GA168 mutant, the space between the commissures is filled with extra axons, as the gaps between them are almost abolished in some segments (yellow arrow) at developmental stage 13–14. (C) Similarly, in C3101 mutant, the space between the commissures is reduced (yellow arrow) at developmental stages 13–14. (D) In H156 mutant, the commissural tracts are abnormally thinner compared to normal embryos at the same stages, stages 13–14. (E) In C022 mutant, the commissural tracts are fused and sharply reduced in thickness during development stages 13–14. (F) In Z1241 mutant, the commissural tracts develop as a single, thin tract without the longitudinal tracts that connect the two commissural tracts per segment at stages 13–14. (G) At developmental stage 14, the commissural tracts are completely separated, and the longitudinal tracts extend anteriorly and posteriorly to join the segments. (H) In GA168, the commissures appear fuzzy in some segments, and the longitudinal tracts thickened at commissural sites. Additionally, these tracts are absent in some segments (blue arrow) at stage 14. (I) In C3101, the commissures exhibit a fuzzy phenotype at developmental stage 14. (J) In H156, the commissural tracts are thickened and fused in some segments (blue arrow) at stage 14. (K) In C022, the commissures develop as a fused, single, thin tract at developmental stage 14. (L) In Z1241, the ladder-like structure of the CNS is disrupted, and the commissures develop as straight, faint lines at the midline, without longitudinal tracts on either side of the midline at developmental stage 14. (M) At developmental stage 15, both CNS axon pathways are fully developed into a ladder-like structure composed of repeated segments. (N) In GA168, [file pone.0333573.s001.tif]

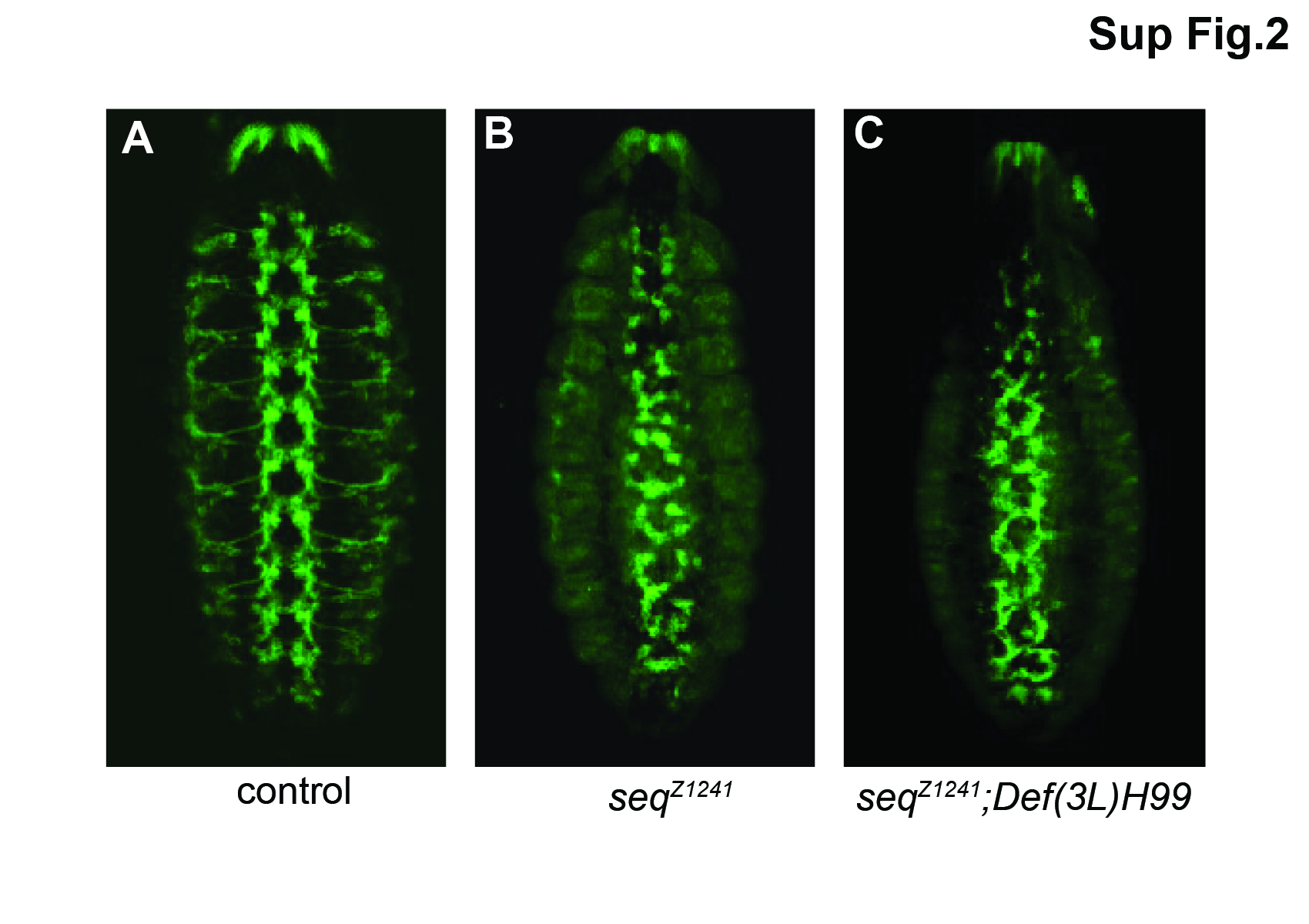

Supplement: S2 Fig — (A) Stage 14 control embryos stained with anti-FasII showing the axonal extensions from the midline; (B) stage 14 seqZ1241 stained with anti-FasII showing the absence of axonal extensions; (C) stage 14 embryos double mutant for seqZ1241 and Def(3L)H99, which removes hid, grim and reaper. (TIF) [file pone.0333573.s002.tif]
