# Supplementary material for: Sequoia affects Drosophila central nervous system development by regulating axonal extension and guidance
Source: PLoS One. 2026 Mar 25;21(3):e0333573. doi: 10.1371/journal.pone.0333573 (PMC13016347; doi:10.1371/journal.pone.0333573)
Supplement: Attachment — Submitted filename: Response to reviewers.docx [file pone.0333573.s005.docx]

**RESPONSE TO REVIEWERS**

**Sequoia affects *Drosophila* central nervous system development by regulating axonal extension and guidance**

Dear editor and reviewer,

We thank the reviewer for his/her comments and suggestions, which will make our manuscript a much better one. Accordingly, we have already made changes to the manuscript (marked in yellow) and some of the figures, and we have performed all the required experiments.

We hope you will find this manuscript suitable for publication at PLOSOne.

Below, we answer the reviewers point by point (in blue).

Reviewer #1: This article by Noor Al-Hajri et al. describes the phenotypes of several new mutant alleles of sequoia (seq), a gene encoding a zinc-finger transcription factor. The study extends previous reports on the role of Sequoia in nervous system development by characterizing five mutant alleles in total—two previously reported and three newly generated. Overall, the phenotypic descriptions are clear and the data are potentially valuable; however, the clarity of presentation and the completeness of the analysis could be improved in several areas.

Major Comments

1. In most figures, only a subset of the alleles is analyzed. Including all five alleles throughout the figures would strengthen the conclusions and provide a more comprehensive view of the phenotypic variability associated with seq mutations.

We have analysed the phenotypes of all five alleles with BP102, 1D4 and 22C10 antibodies. We have included these in the new figures 2 and 3.

2. The manuscript does not specify whether all seq alleles are recessive. No data from heterozygous mutants are shown. Information about dominance or partial dominance could help interpret the phenotypic differences reported, particularly in the final section of the paper.

All alleles analysed are recessive and embryonic lethal, we have included this information in the text.

3. Several statements, especially in the Introduction, lack supporting references. These should be added to properly contextualize the study within previous work on seq and related transcription factors.

We have added the necessary references to the introduction.

4. The discussion does not adequately integrate prior knowledge of seq function. For example, is Seq expressed in all central and peripheral nervous system neurons? Are the reported phenotypes demonstrably cell-autonomous? Addressing these points would clarify how the new alleles relate to the known roles of Sequoia.

We have included this information in the results and discussion section.

5. It would be helpful to include a summary table listing all analyzed phenotypes and the corresponding effects of each allele in the different neuronal types examined.

We have built a summary table (Table 1) with all the alleles, mutations and phenotypes.

Minor Comments

1. Figure formatting

In all figures, the scale bar is too thin and difficult to see. Please increase its thickness. The scale information should remain in the figure legend; currently, it is almost illegible even when zoomed in.

This has been changed.

2. Figure 1G

Ensure that the order of genotypes or conditions in the plot matches the order shown in Figure 1A and in the graphs in Figures 1H–I for easier comparison.

We have reordered the alleles in the graphs.

3. Figures 1H and 1I

Replace “um” with the correct unit symbol “μm”.

This has been changed.

4. Figure 2D

The figure legend lacks information about the antibody used. Moreover, only the mutant is shown. A proper control illustrating the normal phenotype should be included for comparison.

We apologise for this confusion, Fig. 2D shown allele Z1241 stained with 1D4 like all the others. So the control is the same.

5. Figure 3C

The legend should clarify what the light-blue arrow indicates.

We have removed the light blue arrowheads as they were confusing and not showing anything different from the black ones.

6. Figures 4E–G

These panels are not mentioned in the main text; please include a reference to them where appropriate.

This has been changed.

7. Supplementary Figure 1

Add the genotypes directly to the figure to facilitate interpretation. Indicate which antibodies were used in the legend.

This has been changed.

8. line 271

The citation “Figure 5D” should be corrected to “Figure 4D”.

This has been changed.

9. The description of how seq alleles were generated is confusing. The text states that chemical mutagenesis was performed on a second chromosome carrying a fasIII mutation, but it is unclear how the fasIII gene was later reintroduced. Was a genomic FasIII rescue construct inserted into the third chromosome of the mutant flies? Please clarify.

This has been changed.

10. Discussion. Consider whether some of the observed phenotypes could reflect altered cell fate rather than purely axon-guidance defects.

We have included this possibility in the results and discussion section.
