# Supplementary material for: Sequoia affects Drosophila central nervous system development by regulating axonal extension and guidance
Source: PLoS One. 2026 Mar 25;21(3):e0333573. doi: 10.1371/journal.pone.0333573 (PMC13016347; doi:10.1371/journal.pone.0333573)
Supplement: Attachment — Submitted filename: Response to Reviewer Resub2Al-Hajri.docx [file pone.0333573.s006.docx]

We are pleased that the reviewer found the paper to be of interest and worthy of publication. We have addressed the two minor issues raised by the referee in response to our resubmission of the manuscript.

1. “The way in which the fasIII gene rescue was performed is still unclear.”

- we have added further information and provided a reference to the stock that was used to perform the fasIII rescue on page 4.

2. There is a misreferenced figure in the section describing the mutant alleles in Figure 2. The authors cite Figure 2K′ when referring to motor axonal defects, but I believe they intended to cite Figure 2M′.

- we have corrected this citation on page 8 to cite Figure 2M’

Thank you for considering our work.

Yours sincerely,

Guy Tear and Sofia J. Araújo
